# Supplementary material for: Detection of Extremely Low Level Ciguatoxins through Monitoring of Lithium Adduct Ions by Liquid Chromatography-Triple Quadrupole Tandem Mass Spectrometry
Source: Toxins (Basel). 2024 Mar 27;16(4):170. doi: 10.3390/toxins16040170 (PMC11053878; doi:10.3390/toxins16040170)
Supplement: Supplementary file 1 [file toxins-16-00170-s001.zip › toxins-2655454-supplementary.pdf]

# Supplementary Material

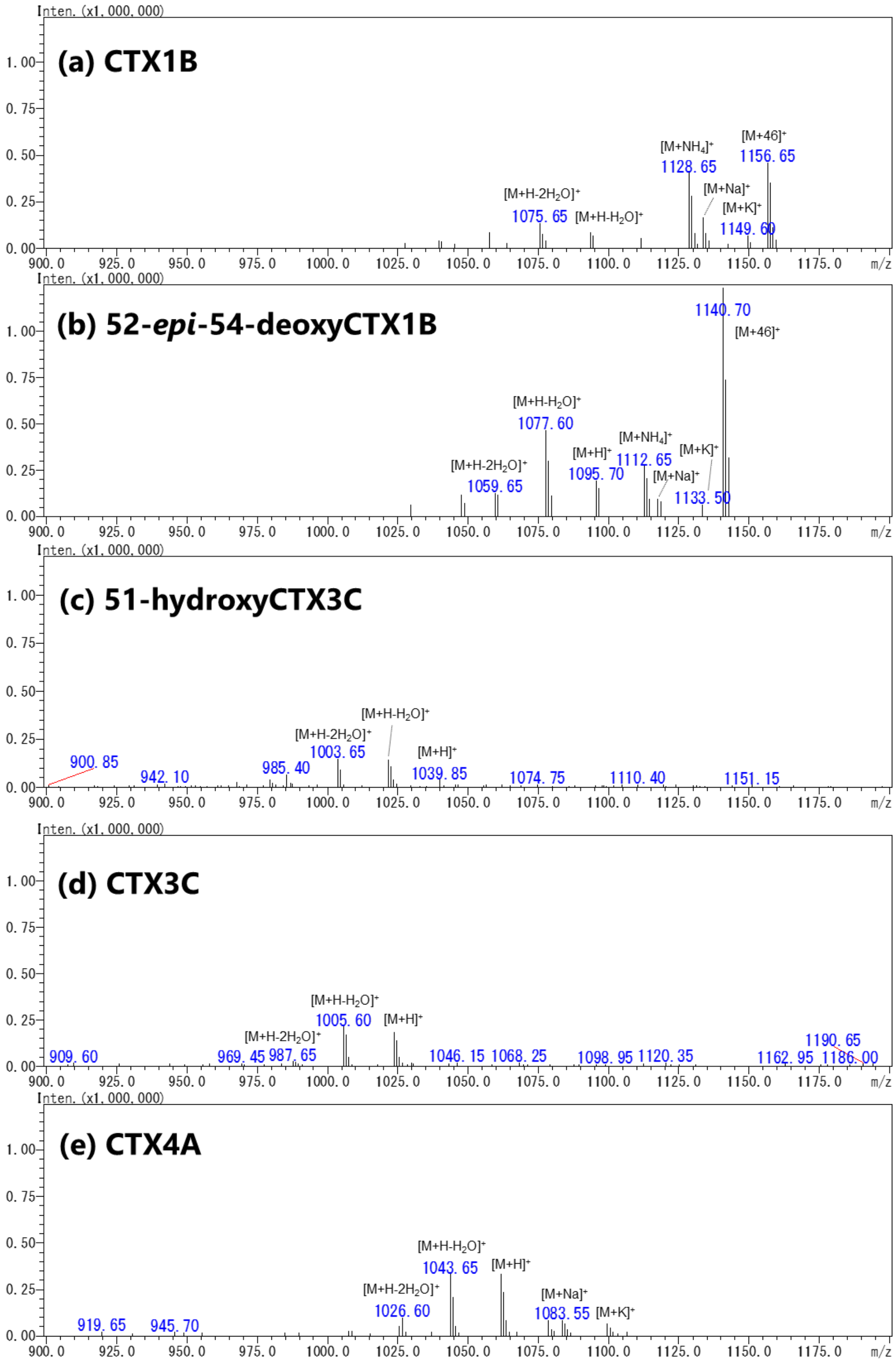


**Figure S1. A.** Mass spectra of CTXs observed by Scan mode under the acetonitrile mobile phase condition. (**a**) CTX1B, (**b**) 52-*epi*-54-deoxyCTX1B, (c) 51-hydroxyCTX3C, (d) CTX3C, (e) CTX4A.

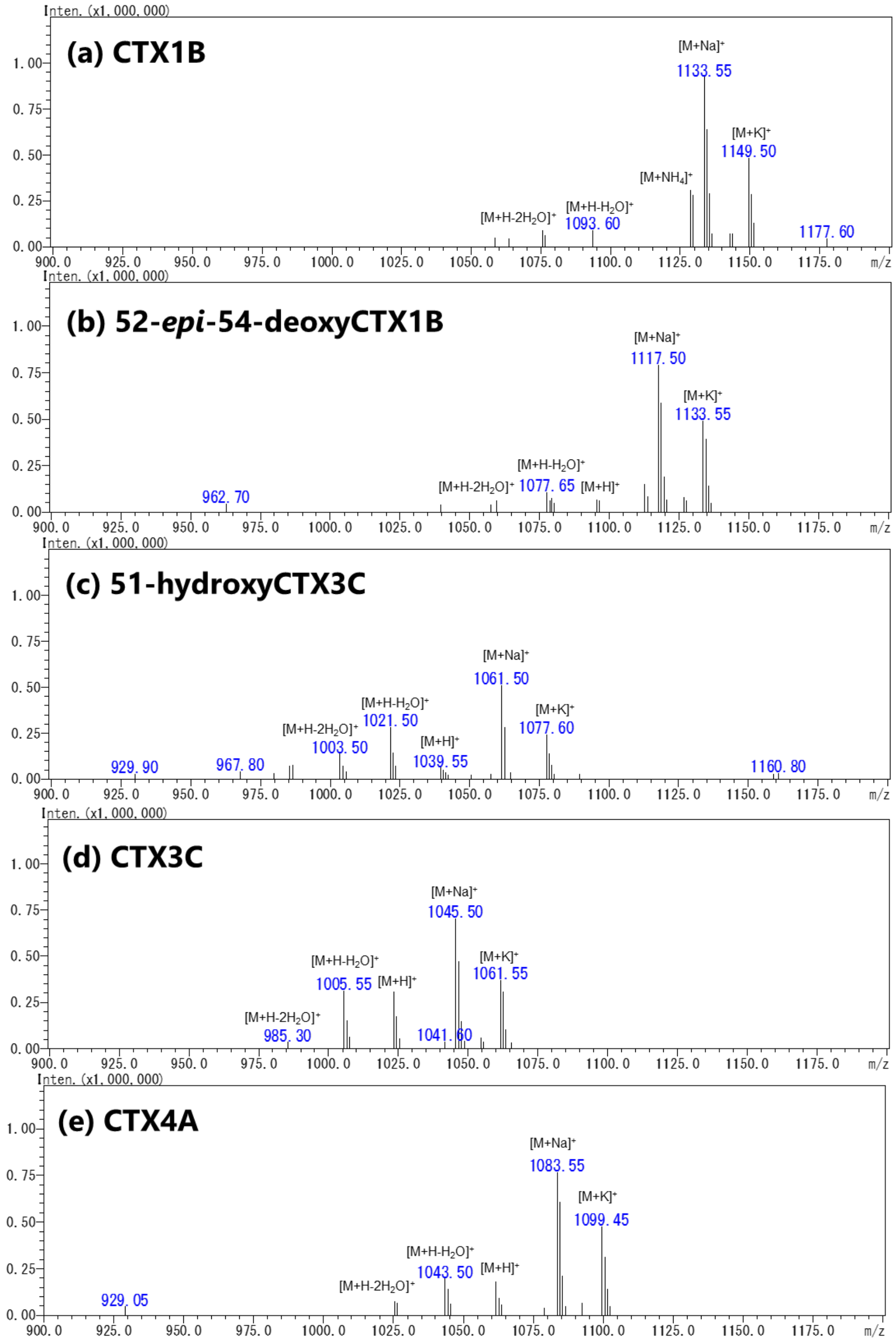


**Figure S1B.** Mass spectra of CTXs observed by Scan mode under the methanol mobile phase condition. (**a**) CTX1B, (**b**) 52-*epi*-54-deoxyCTX1B, (c) 51-hydroxyCTX3C, (d) CTX3C, (e) CTX4A.

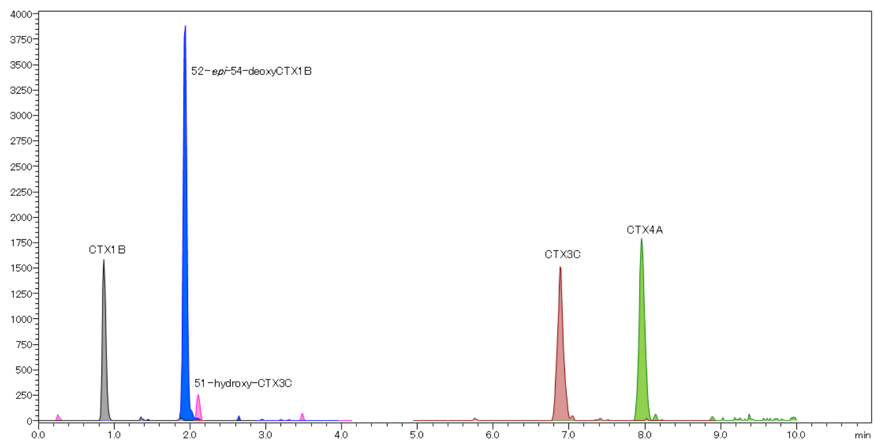


**(a)**

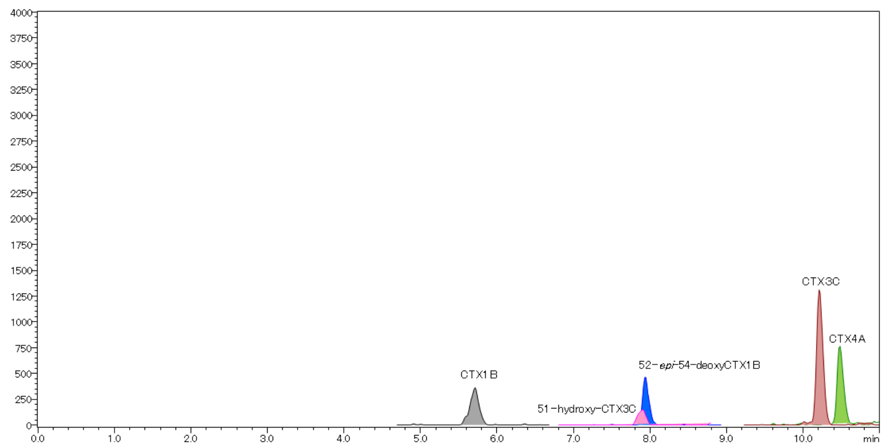


**(b)**

**Figure S2.** The LC-MS/MS (MRM) chromatograms of CTXs recording under the conditions described in previous reports (The transitions and collision energy were optimized to give best sensitivity for each analog. CTX1B: *m/z* 1128.60>1093.60 and CE -21.0 V, 51-hydroxy-CTX3C: *m/z* 1056.60>1039.60 and CE -15.0 V, 52-*epi*-54-deoxyCTX1B: *m/z* 1112.60>1077.60 and CE -22.0 V, CTX3C: *m/z* 1023.60>125.10 and CE -48.0 V, CTX4A: *m/z* 1078.60>1043.60 and CE; -19.0 V). (a) acetonitrile-based mobile phases [40]. (b) methanol-based mobile phases[10].

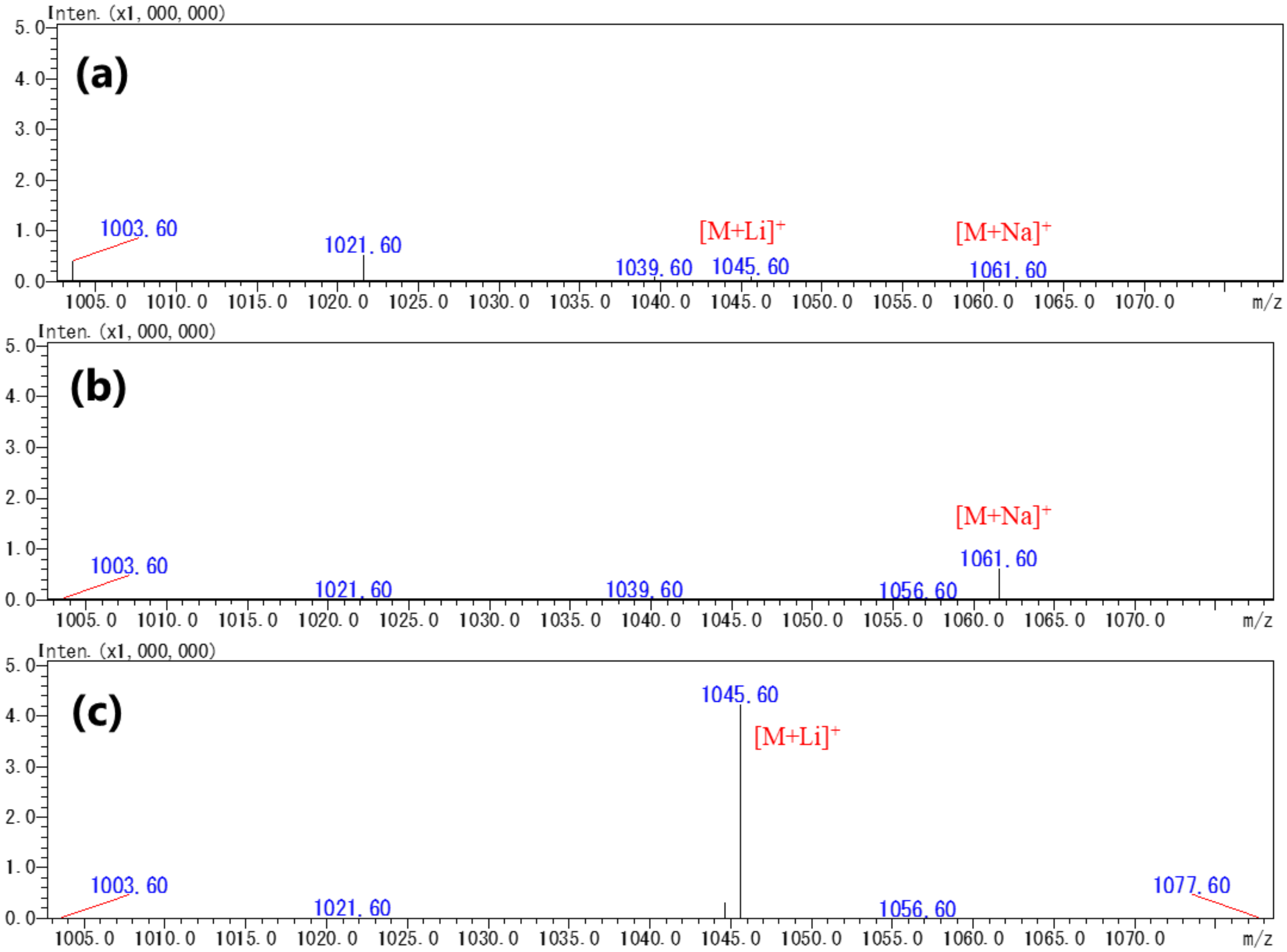


**Figure S3.** Mass spectra of 51-hydroxyCTX3C taken under the acetonitrile-based mobile phases (a) without any supplement, (b) supplemented with $Na^+$ (NaOH), and (c) supplemented with $Li^+$ (LiOH).

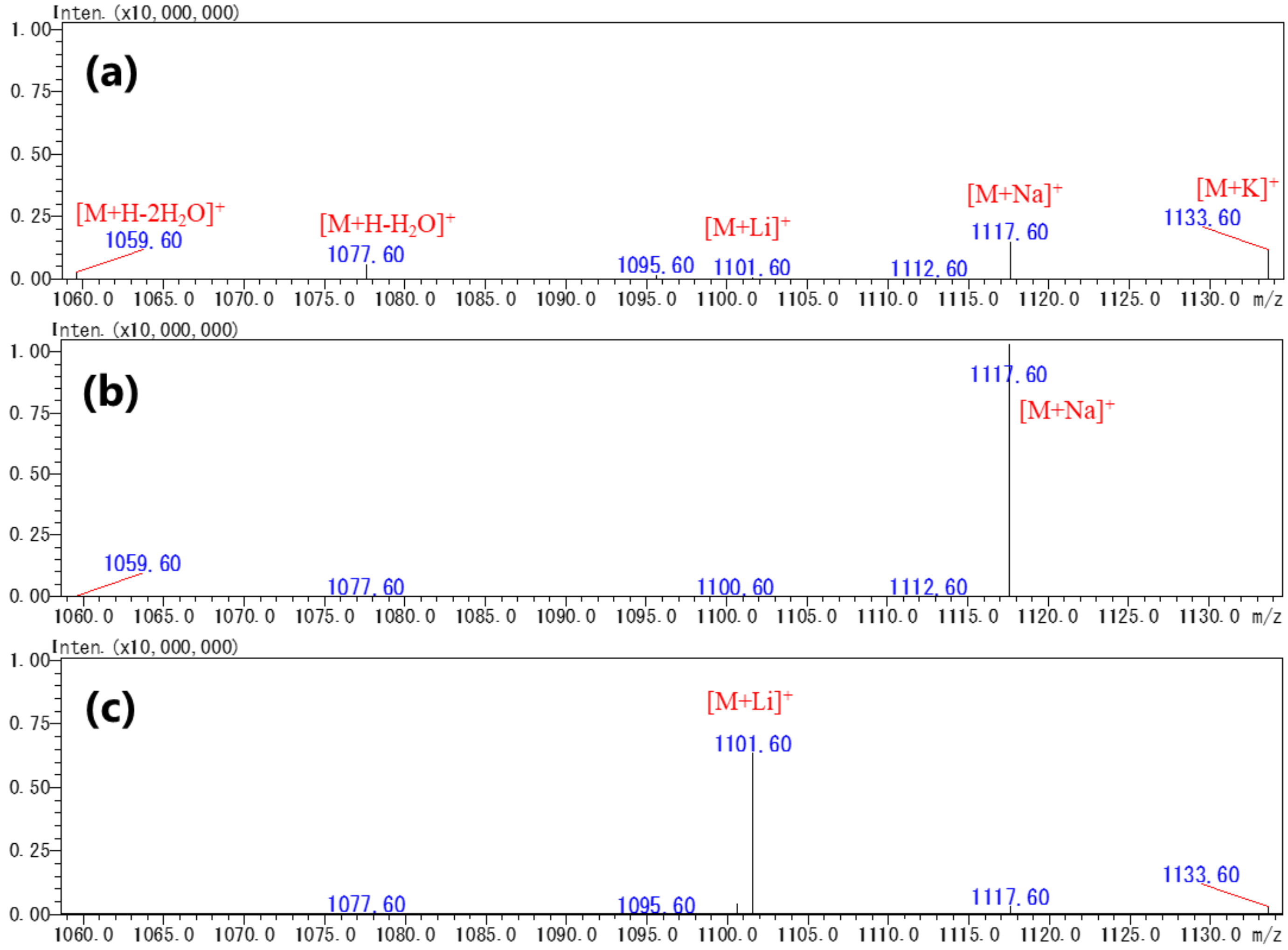


**Figure S4.** Mass spectra of 52-*epi*-54-deoxyCTX1B taken under the acetonitrile-based mobile phases (a) without any supplement, (b) supplemented with $Na^+$ (NaOH), and (c) supplemented with $Li^+$ (LiOH).

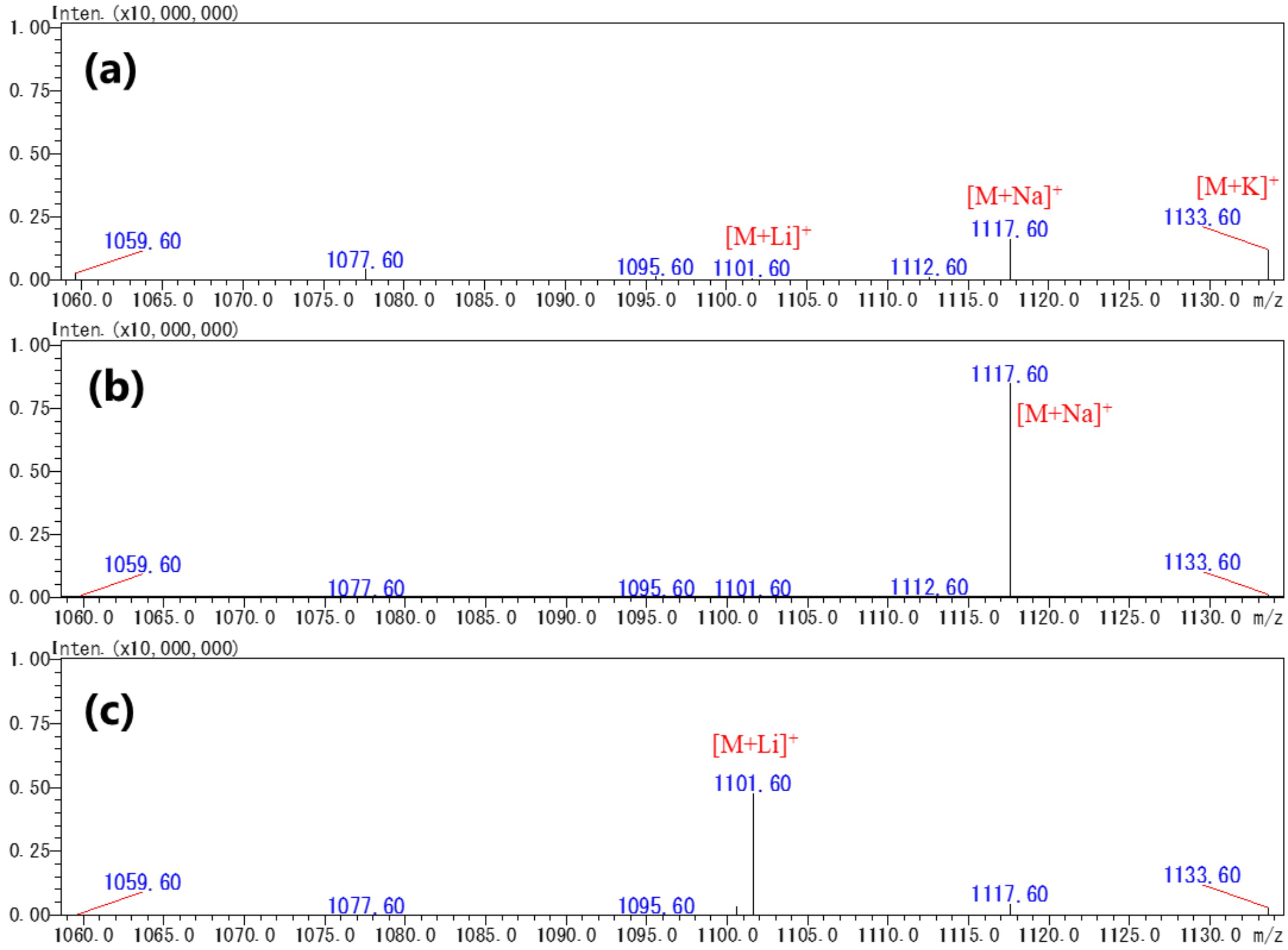


**Figure S5.** Mass spectra of 54-deoxyCTX1B taken under the acetonitrile-based mobile phases (a) without any supplement, (b) supplemented with $Na^+$ (NaOH), and (c) supplemented with $Li^+$ (LiOH).

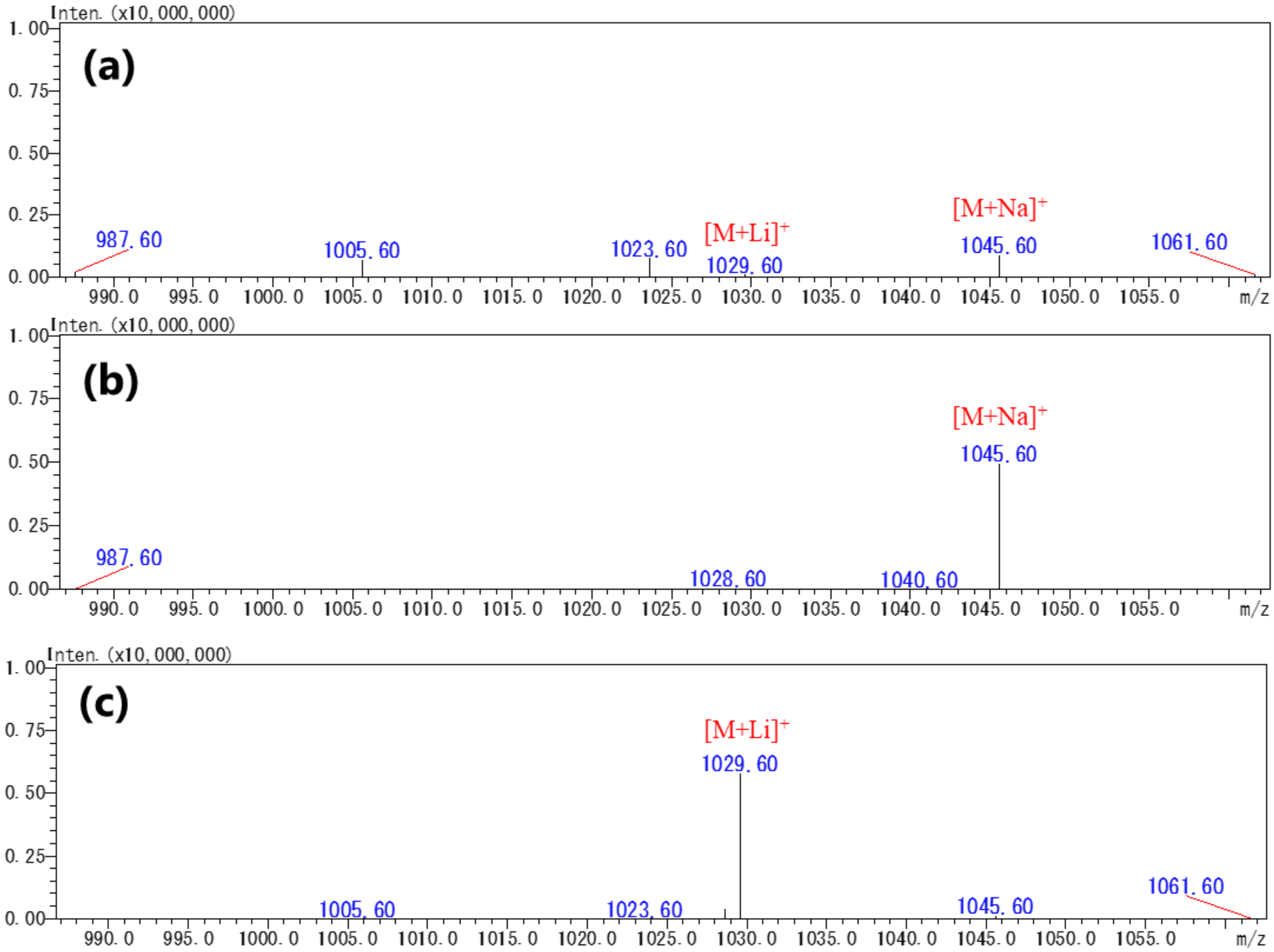


**Figure S6.** Mass spectra of 49-*epi*CTX3C taken under the acetonitrile-based mobile phases (a) without any supplement, (b) supplemented with $Na^+$ (NaOH), and (c) supplemented with $Li^+$ (LiOH).

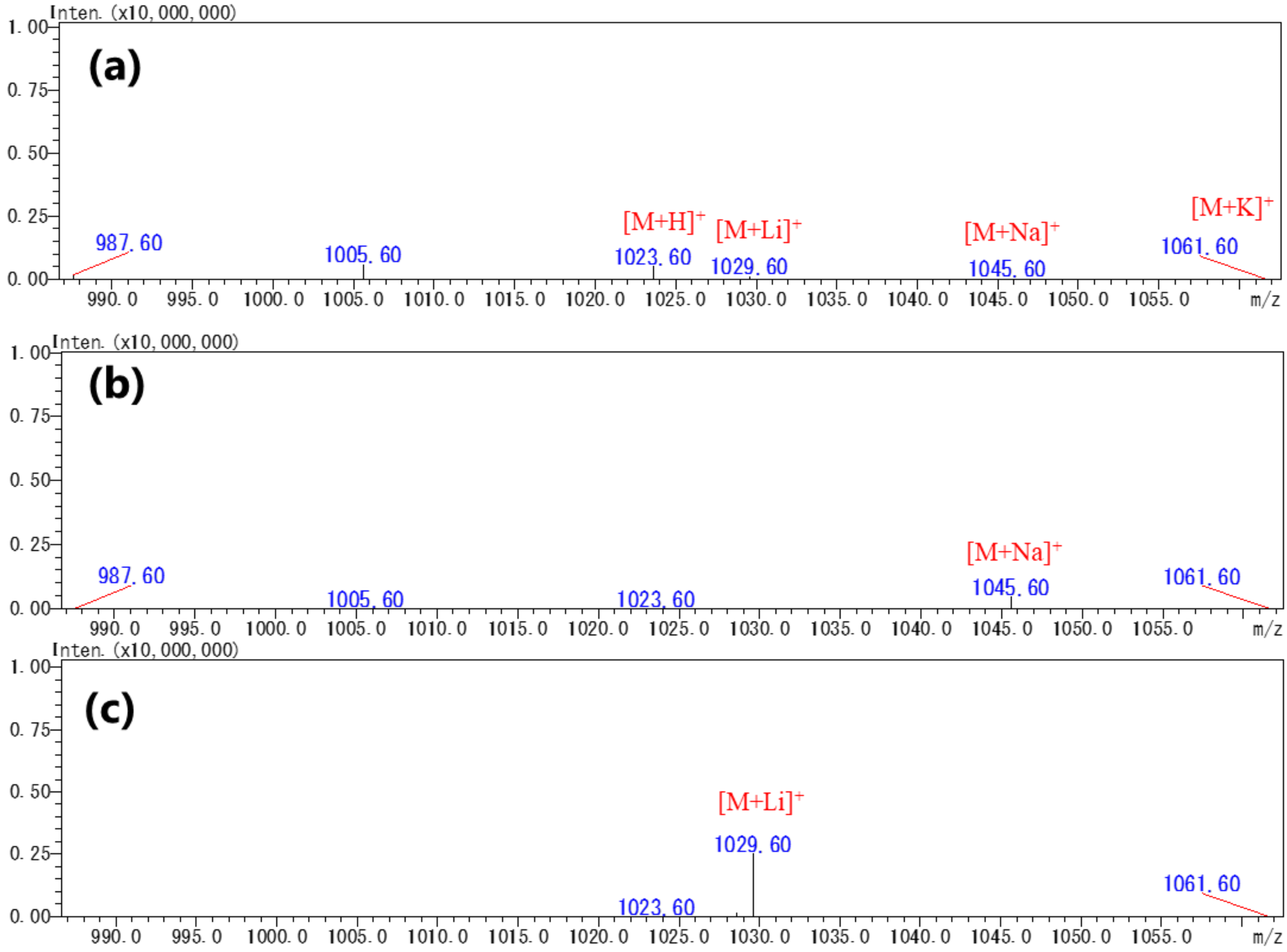


**Figure S7.** Mass spectra of CTX3C taken under the acetonitrile-based mobile phases (a) without any supplement, (b) supplemented with $Na^+$ (NaOH), and (c) supplemented with $Li^+$ (LiOH).

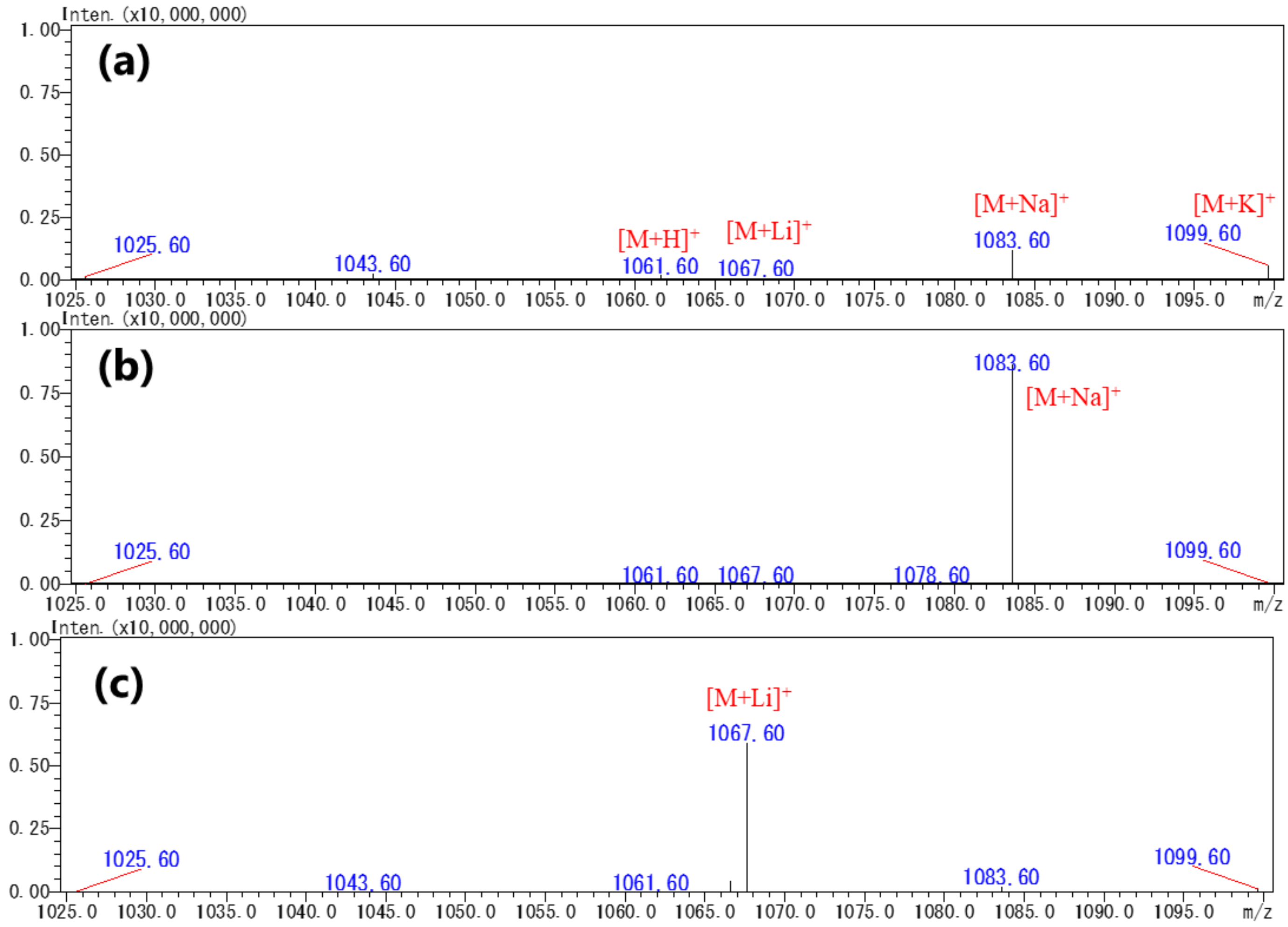


**Figure S8.** Mass spectra of CTX4A taken under the acetonitrile-based mobile phases (a) without any supplement, (b) supplemented with $Na^+$ (NaOH), and (c) supplemented with $Li^+$ (LiOH).

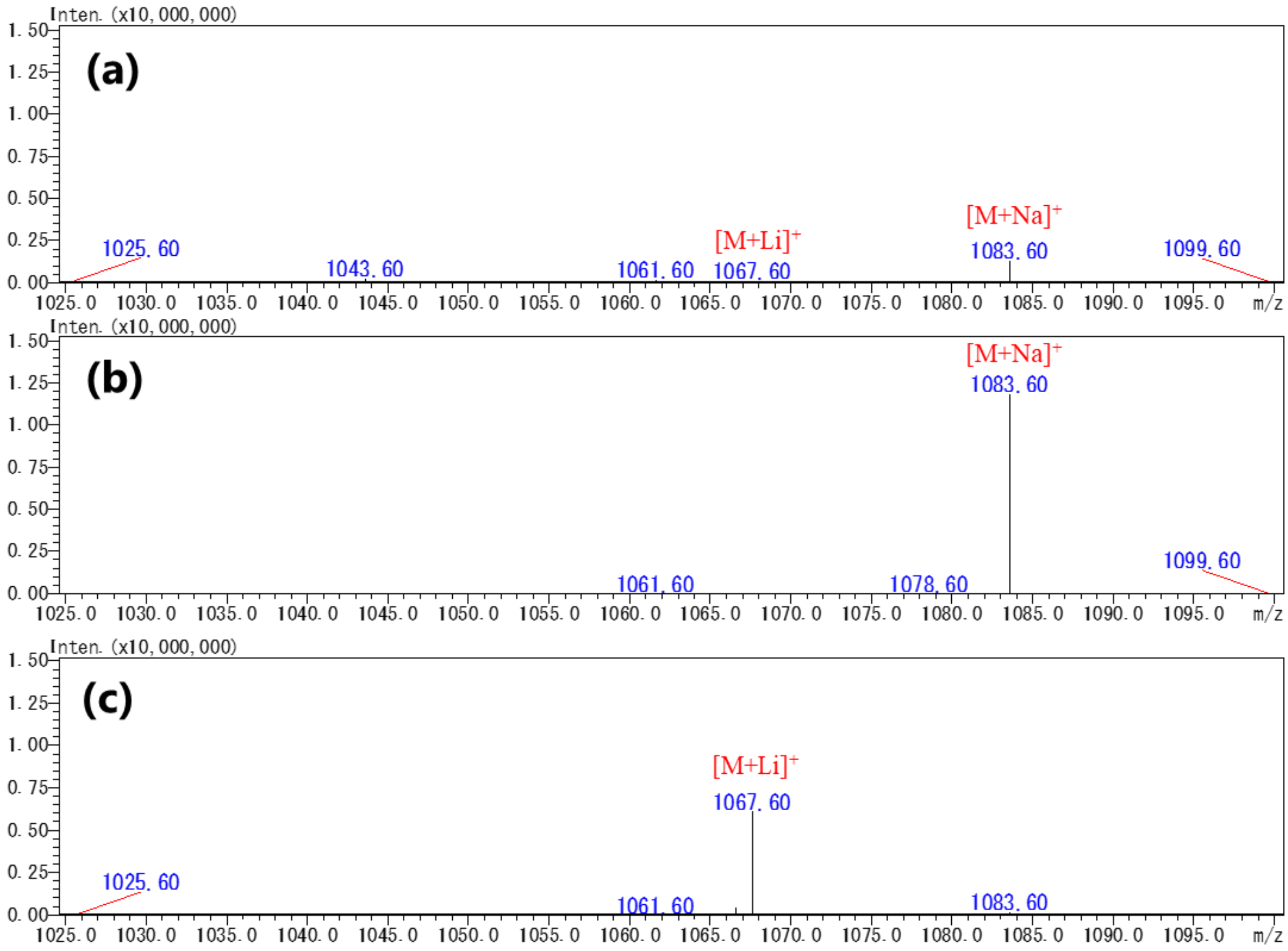


**Figure S9.** Mass spectra of CTX4B taken under the acetonitrile-based mobile phases (a) without any supplement, (b) supplemented with $Na^+$ (NaOH), and (c) supplemented with $Li^+$ (LiOH).
